# Association between the telecommuting environment and somatic symptoms among teleworkers in Japan

**DOI:** 10.1093/joccuh/uiad014

**Published:** 2023-12-19

**Authors:** Satoru Kanamori, Takahiro Tabuchi, Yuko Kai

**Affiliations:** Graduate School of Public Health, Teikyo University, Tokyo, 173-8605, Japan; Department of Preventive Medicine and Public Health, Tokyo Medical University, Tokyo, 160-8402, Japan; Cancer Control Center, Osaka International Cancer Institute, Osaka, 541-8567, Japan; Physical Fitness Research Institute, Meiji Yasuda Life Foundation of Health and Welfare, Tokyo, 192-0001, Japan

**Keywords:** teleworking, home environment, somatic symptoms, occupational health

## Abstract

**Objectives:** This study aimed to clarify the association between telecommuting environments and somatic symptoms among teleworkers in Japan.

**Methods:** This cross-sectional study, conducted from September 27 to October 29, 2021, used data from the Japan COVID-19 and Society Internet Survey (JACSIS study) in Japan. Of the 31 000 male and female respondents, who were Japanese residents aged 15-79 years and were randomly selected from the panel members of an internet survey company, 4569 home-based teleworkers were finally included in the analysis; 26 431 respondents who met the exclusion criteria were excluded. The analysis included 4 cut-offs (≥4, 8, 12, and 16 points) for somatic symptoms on the Somatic Symptom Scale-8 as objective variables, and the telecommuting environment, such as having adequate desk light and a quiet environment, as explanatory variables. Adjusted Poisson regression analysis was conducted using demographic variables as covariates.

**Results:** The prevalence ratio (PR) for somatic symptoms increased significantly as the number of poor telecommuting conditions increased, regardless of the cut-off value for somatic symptoms or the frequency of teleworking. In the telecommuting environment, the PR for somatic symptoms was significantly higher for the following 6 items: poor teleworking space to concentrate, inadequate foot space, poor communication environment, poor space for relaxation, noise, and inappropriate temperature and humidity.

**Conclusions:** These results suggest that for home-based teleworkers, the more inadequate the telecommuting environment, especially in the aforementioned 6 areas, the higher the likelihood of somatic symptoms. Improving these environments may be useful in preventing various somatic symptoms.

## Teaser

**Key points:**

- **What is already known on this topic:** The prevalence of teleworking experienced a notable surge amidst the COVID-19 pandemic. Inappropriate and inadequate telecommuting settings may result in the manifestation of somatic symptoms, namely musculoskeletal discomfort and psychological distress. The degree of satisfaction associated with teleworking environments exhibits an inverse correlation with the occurrence of health-related complications, such as musculoskeletal ailments and cardiovascular disorders.
- **What this study adds:** The more inadequate the telecommuting environment, the higher the likelihood of somatic symptoms, regardless of the cut-off for somatic symptoms or the frequency of telecommuting. Six specific factors can exacerbate somatic symptoms in a telecommuting environment: poor teleworking space to concentrate, inadequate foot space, poor communication environment, poor space for relaxation, noise, and inappropriate temperature and humidity.
- **How this study might affect research, practice, or policy:** Future longitudinal and intervention studies are needed to clarify these causal relationships. In practice, encouraging workers to maintain appropriate telecommuting environments and companies to support such environments may help prevent somatic symptoms.

## 1. Introduction

Teleworking from home has spread rapidly during the coronavirus disease 2019 (COVID-19) pandemic. During the 2020 lockdown, 47% of workers teleworked in Australia, France, and the United Kingdom.^1^ In Japan, teleworking was at 10% in December 2019 before the COVID-19 pandemic, but rose to 28% in May 2020 after the start of the pandemic. Although the COVID-19 emergency phase ended in May 2023,^2^ teleworking is expected to continue to some extent, even after the pandemic.^3^

Teleworking has some advantages, such as increased flexibility and autonomy, reduced commuting time, and increased productivity, as well as disadvantages, such as a lack of support, inadequate equipment, and presenteeism.^4^ Several systematic reviews have reported that the telecommuting environments are inadequately developed^5^^,^^6^; an inadequate physical environment^7^ and computer workstation design in the office^8^ can cause somatic symptoms such as eyestrain and musculoskeletal pain. Therefore, the risk of somatic symptoms may be increased in poor telecommuting environments, including inadequate physical environment, ergonomic resources, and organizational factors (eg, support for telework) related to telework.^9^^,^^10^

Several studies have examined the association between teleworking environments and somatic symptoms among teleworkers. A cross-sectional study of Japanese workers showed that those working in environments with inadequate desk light, foot space, and temperature/humidity control had a higher prevalence of shoulder pain than those who did not.^11^ Lack of a dedicated workplace to concentrate on work and inadequate desk workspace are associated with a higher prevalence of low back pain.^12^ Inadequate air quality and humidity may also cause eye and respiratory irritations, headaches, and fatigue, whereas inadequate communication environments and other factors may cause stress reactions.^10^ Based on these findings, it is assumed that the telecommuting environment is associated with several somatic symptoms.

To the best of our knowledge, few studies have investigated this association. A cross-sectional study examining the association between telecommuting environments and physical health reported that satisfaction with various telecommuting environments was inversely associated with several health problems (eg, musculoskeletal, cardiovascular).^13^ However, no previous study has clarified the relationship between the telecommuting environment and somatic symptoms.

Therefore, this study aimed to clarify the association between telecommuting environments and somatic symptoms among teleworkers in Japan. Furthermore, a previous cross-sectional study reported that those with a good telecommuting environment showed no association with low back pain despite frequent telework, whereas the odds ratio was higher for those with a poor telecommuting environment as the frequency of teleworking increased.^14^ Because it is suggested that the association may differ depending on the frequency of teleworking, this study aimed to examine this association by the frequency of teleworking.

## 2. Materials and methods

### 2.1. Study design and setting

This cross-sectional study used data from an internet survey in the Japan COVID-19 Issue-based Assessment of Social and Health Disparities Study (JACSIS: The Japan COVID-19 and Society Internet Survey).^15^ The JACSIS is a web-based self-reporting questionnaire survey conducted by a major internet research firm (Rakuten Insight Corporation, Tokyo, Japan).

### 2.2. Participants

The survey, conducted from September 27 to October 29, 2021, targeted Japanese residents of both sexes, aged 15-79 years, from among the panel members of Rakuten Insight, Inc., an internet research company. In line with Japan’s population distribution, panel members were randomly sampled by sex, age, and prefecture and then requested to respond to the survey.

Of the 31 000 valid survey responses, a total of 2825 respondents were excluded from the analysis for inappropriate or incorrect responses, including those who selected all of the survey items related to drug use; those who selected all of the survey items related to drug use or chronic disease; and those who did not select the appropriate item for the question, “Please select the second last option from the following choices.” Furthermore, an additional 23 606 persons were excluded as follows: non-workers (students not working, retirees, housewives/househusbands, and the unemployed), because this study was conducted among home-based teleworkers; those who worked zero hours in the previous month among those who worked; those who did not telework among those who worked; and those who worked on days when they teleworked in the previous month (average hours worked) among those who worked none (zero hours). The total number of participants finally included in the analysis was 4569.

### 2.3. Measures

#### 2.3.1. Somatic symptoms

The Japanese version of the Somatic Symptom Scale-8 (SSS-8), with verified reliability and validity, was used to evaluate the somatic symptoms.^16^ This scale consists of 8 questions on the following issues: (1) stomach or bowel problems, (2) back pain, (3) pain in the arms, legs, or joints, (4) headaches, (5) chest pain or shortness of breath, (6) dizziness, (7) fatigue or lethargy, and (8) insomnia. Respondents rate the extent to which each symptom has bothered them during the previous 7 days and score each item from 0 to 4 according to the following: “Not at all (0 points),” “A little bit (1 point),” “Somewhat (2 points),” “Quite a bit (3 points),” and “Very much (4 points),” and the total score is calculated. Based on the total score, participants were classified into 5 severity groups. Cut-offs for the total score according to severity were ≥4 points (low severity), ≥8 points (medium severity), ≥12 points (high severity), and ≥16 points (very high severity). Therefore, the analysis was conducted by dividing the data into 4 patterns. For the Poisson regression analysis, the following outcomes were used: 0 = no somatic symptoms and 1 = somatic symptoms.

#### 2.3.2. Telecommuting environment

Regarding the telecommuting environment, 14 items were selected based on previous research^12^^,^^14^ and guidelines on teleworking from the Japanese Ministry of Health, Labor, and Welfare.^17^ Specifically, these included the following: (1) Do you have a place or room where you can concentrate on your work? (a place or room to concentrate); (2) Do you have enough light on the desk for you to work? (sufficient desk light); (3) Do you have enough space on your desk to work? (sufficient workspace on the desk); (4) Do you have enough foot space under your desk to stretch your legs? (sufficient foot space); (5) Are the temperature and humidity conditions in your workroom comfortable? (comfortable temperature and humidity); (6) Do you have a quiet environment for work, ie, no traffic noise, noises from daily life, etc (quiet environment); (7) Did you receive any financial assistance in developing a telework work environment, eg, assistance in purchasing desks, chairs, computer equipment, etc (financial support for work environment improvement)”; (8) Did you receive any advice or guidance from your workplace regarding the teleworking environment and methods? (advice and guidance from the workplace); (9) Do you use an office desk or chair, including children’s study desks (use of an office desk and chair); (10) Do you use a low table or kotatsu to work? (use of a low table or kotatsu); (11) Do you work at a standing desk? (use of a standing desk); (12) Do you have a stable communication environment such as an internet connection? (stable communication environment); (13) Are your PCs and other communication devices performing adequately? (adequate performance of communication devices); and (14) Do you have a place or environment where you can change your mood or refresh yourself? (a place or environment to refresh oneself). For all these questions, the options were, “Yes,” “Somewhat yes,” “Somewhat no,” and “No.”

Two methods were used for the telecommuting environment in the analysis. The first was the number of poor telecommuting environments, and the second was the telecommuting environment (14 types). In both methods, “yes” and “somewhat yes” were treated as “yes,” and “somewhat no” and “no” were treated as “no.” In addition, “yes” was regarded as a good telecommuting environment and “no” as a poor telecommuting environment. However, only the question “Do you use a low table or kotatsu to work?” treated the response “yes” as a poor telecommuting environment. The number of poor telecommuting environments was the number of items corresponding to the response “No” (minimum 0, maximum 14). The number of poor telecommuting environments was 0 for 33 (0.7%), 10 for 156 (3.4%), 11 for 97 (2.1%), 12 for 80 (1.8%), 13 for 69 (1.5%), and 14 for 12 (0.3%) respondents. Owing to the small numbers in these categories, aggregate categories of “0-1” and “≥10” were created.

#### 2.3.3. Telecommuting frequency

The frequency of telecommuting in the past month was asked, and the options were never, once a month, 2-3 times a month, once a week, 2-3 times a week, 4-5 times a week, or almost every day (to 6-7 times a week). Those who answered “never” were treated as those who did not telecommute and were excluded from the analysis.

#### 2.3.4. Other covariates

The following were used as covariates: sex (male, female), age (16-19, 20-24, 25-29, 30-34, 35-39, 40-44, 45-49, 50-54, 55-59, 60-64, 65-69, 70-74, and ≥75 years), body mass index (BMI, <18.5, 18.5-25.0, and ≥25.0), smoking (never, yes), alcohol consumption (less frequent than daily, more than daily), physical activity (no, yes), mental health (K6)^18^ (good, poor), household income (<4 million yen, 4-8 million yen, ≥8 million yen, and don’t want to answer/don’t know), education (junior high school, high school, vocational school, junior college, technical college, university, graduate school, and other), type of industry (primary industry, secondary industry, and tertiary industry), working hours (<40 h/wk, >40 h/wk), and company size (≤49 or fewer employees, 50-999, ≥1000).

### 2.4. Statistical analysis

Poisson regression analysis was conducted with somatic symptoms as the objective variable; telecommuting environments as the explanatory variable; and sex, age, BMI, smoking, alcohol consumption, physical activity, mental health, household income, education, type of industry, working hours, company size, and telecommuting frequency as covariates. The choice of Poisson regression for analysis was because the proportion of participants for each outcome in this study exceeded 10%, and as such, the odds ratios calculated by logistic regression analysis would deviate from the actual risk ratios.^19^^,^^20^

In the Poisson regression analysis, the “0-1” of the number of poor telecommuting environments was used as a reference, and the prevalence ratio (PR) was calculated for each category. For trend testing, the number of poor telecommuting environments was treated as a continuous variable. One item was entered into the model for each telecommuting environment. Additionally, in this analysis, a poor telecommuting environment was used as the reference.

In addition, whether the association between telecommuting environments and somatic symptoms differed according to the telecommuting frequency was determined. As the median frequency of teleworking was 2-3 times a week, the participants were divided into 2 groups: those who did 3 or fewer times per week and those who did 4 or more times per week.

## 3. Results

Table 1 shows the characteristics of the participants according to telecommuting frequency. Overall, 3109 (68.0%) participants were men, with a mean age of 43.8 years (SD, 14.0). A total of 2933 (64.2%) worked at home 3 or fewer times per week, and 1636 (35.8%) worked 4 or more times per week. In the group that worked at home 3 or fewer times per week, 824 (28.1%) respondents scored ≤3 with few somatic symptoms, whereas in the group that worked at home 4 or more times per week, 562 (34.4%) showed higher scores.

**Table 1:** Characteristics of teleworkers by frequency of telecommuting.

		**Frequency of telecommuting**					
		**≤3 d/wk (*N* = 2933)**		**≥4 d/wk (*N* = 1636)**		**Overall (*N* = 4569)**	
		***n***	**%**	***n***	**%**	***n***	**%**
**Sex**	**Male**	2029	69.2	1080	66.0	3109	68.0
**Age, y**	**Mean, SD**	42.5	14.0	46	13.7	43.8	14.0
**BMI**	**<18.5**	316	10.8	184	11.2	500	10.9
	**18.5-24.9**	2080	70.9	1079	66.0	3159	69.1
	**≥25.0**	537	18.3	373	22.8	910	19.9
**Lifestyle behavior**	**Non-smoker**	2144	73.1	1271	77.7	3415	74.7
	**Frequency of drinking less than daily**	2386	81.4	1275	77.9	3661	80.1
	**Being physically active**	1328	45.3	617	37.7	1945	42.6
**Mental health**	**Poor**	1639	55.9	1019	62.3	2658	58.2
**Annual household income**	**<4.0 million yen**	441	15.0	297	18.2	738	16.2
	**4.0-7.9 million yen**	1101	37.5	518	31.7	1619	35.4
	**≥8.0 million yen**	1011	34.5	540	33.0	1551	33.9
	**Unknown/undisclosed**	380	13.0	281	17.2	661	14.5
**Education**	**Junior high/high school**	348	11.9	207	12.7	555	12.1
	**Vocational school/junior College/technical college**	363	12.4	253	15.5	616	13.5
	**University/college**	1872	63.8	949	58.0	2821	61.7
	**Graduate school**	338	11.5	212	13.0	550	12.0
	**Other**	12	0.4	15	0.9	27	0.6
**Type of industry**	**Primary industry**	23	0.8	8	0.5	31	0.7
	**Secondary industry**	798	27.2	400	24.4	1198	26.2
	**Tertiary industry**	2112	72.0	1228	75.1	3340	73.1
**Working hours**	**<40 h/wk**	1072	36.5	630	38.5	1702	37.3
	**≥40 h/wk**	1861	63.5	1006	61.5	2867	62.7
**Company size (number of employees)**	**<50**	660	22.5	653	39.9	1313	28.7
	**50-999**	964	32.9	334	20.4	1298	28.4
	**≥1000**	1165	39.7	559	34.2	1724	37.7
	**Unknown**	144	4.9	90	5.5	234	5.1
**Frequency of telecommuting**	**1/mo**	425	14.5	0	0.0	425	9.3
	**2-3/mo**	656	22.4	0	0.0	656	14.4
	**1/wk**	662	22.6	0	0.0	662	14.5
	**2-3/wk**	1190	40.6	0	0.0	1190	26.0
	**4-5/wk**	0	0.0	725	44.3	725	15.9
	**Almost every day (6-7/wk)**	0	0.0	911	55.7	911	19.9
**Somatic symptoms**	**<4**	824	28.1	562	34.4	1386	30.3
	**4-7**	723	24.7	415	25.4	1138	24.9
	**8-11**	511	17.4	322	19.7	833	18.2
	**12-15**	423	14.4	166	10.1	589	12.9
	**≥16**	452	15.4	171	10.5	623	13.6

BMI, body mass index.

Table 2 presents the results of the Poisson regression analysis of the association between the number of poor telecommuting environments and somatic symptoms. No explanatory variables or covariates had a strong correlation (Spearman’s rank correlation coefficient: −0.258 to 0.266). For all cut-offs for somatic symptoms, the PR increased significantly as the number of poor telecommuting environments increased. The analysis using a cut-off of 4 points (low severity) for somatic symptoms showed that the PR was significantly high for all cut-offs of 2 or more poor telecommuting environments; the PR was significantly higher for all cut-offs when the number of poor telecommuting environments was 6 or more.

**Table 2:** Prevalence ratios of somatic symptoms according to the number of poor telecommuting environments.

**Number of poor telecommuting environments**		**Somatic symptoms (the cut-off point)**											
		**Low (≥4)**			**Medium (≥8)**			**High (≥12)**			**Very high (≥16)**		
	***n***	**PR**	**95% CI**	***P***	**PR**	**95% CI**	***P***	**PR**	**95% CI**	***P***	**PR**	**95% CI**	***P***
0-1	458	ref.			ref.			ref.			ref.		
2	463	**1.20**	**1.08-1.34**	**<.001**	1.15	0.97-1.36	.110	**1.44**	**1.10-1.87**	**.007**	1.13	0.72-1.77	.600
3	774	**1.17**	**1.06-1.30**	**.002**	1.05	0.90-1.23	.513	0.91	0.69-1.19	.488	0.75	0.48-1.17	.208
4	617	**1.37**	**1.25-1.52**	**<.001**	**1.30**	**1.11-1.51**	**<.001**	1.23	0.95-1.60	.117	1.10	0.73-1.68	.640
5	534	**1.37**	**1.24-1.51**	**<.001**	**1.27**	**1.09-1.48**	**.002**	**1.50**	**1.17-1.92**	**.002**	1.30	0.86-1.94	.211
6	452	**1.43**	**1.30-1.58**	**<.001**	**1.51**	**1.30-1.74**	**<.001**	**1.84**	**1.44-2.34**	**<.001**	**1.75**	**1.19-2.56**	**.004**
7	357	**1.39**	**1.26-1.53**	**<.001**	**1.44**	**1.24-1.66**	**<.001**	**1.80**	**1.42-2.29**	**<.001**	**1.94**	**1.34-2.81**	**<.001**
8	268	**1.43**	**1.30-1.58**	**<.001**	**1.44**	**1.24-1.68**	**<.001**	**1.77**	**1.39-2.26**	**<.001**	**1.97**	**1.35-2.89**	**<.001**
9	232	**1.43**	**1.30-1.59**	**<.001**	**1.55**	**1.33-1.80**	**<.001**	**2.07**	**1.62-2.64**	**<.001**	**2.32**	**1.58-3.40**	**<.001**
10 or more	414	**1.37**	**1.24-1.51**	**<.001**	**1.48**	**1.28-1.70**	**<.001**	**2.08**	**1.65-2.62**	**<.001**	**2.65**	**1.85-3.79**	**<.001**
		*P* for trend		**<.001**	*P* for trend		**<.001**	*P* for trend		**<.001**	*P* for trend		**<.001**

The PR was calculated for each cut-off for somatic symptoms (“Low” PR was classified as 0-3 points and 4-32 points, and PR of 4 or more points was calculated). Adjusted for sex, age, lifestyle behaviors (smoking, drinking alcohol, and physical activity), mental health, annual household income, education, type of industry, working hours, company size, and frequency of telecommuting. Bolded figures indicate significant differences. PR, prevalence ratio.

Appendix 1 presents the results of the same analysis as in Table 2 for the frequency of teleworking. The PR was significantly higher for all teleworking frequencies and cut-offs for somatic symptoms as the number of poor teleworking environments increased. For those who teleworked 3 or fewer times per week, the PR was significantly higher at all cut-offs for somatic symptoms when the number of poor teleworking environments was 6 or more. Similarly, for the group that teleworked 4 or more times per week, the PR was significantly higher at all cut-offs for somatic symptoms when the number of poor teleworking environments was 9 or more.

Table 3 presents the results of the Poisson regression analysis of the association between the teleworking environment and somatic symptoms. Using cut-offs of 4, 8, 12, and 16 points for increasing severity of somatic symptoms, an association was found for 11, 10, 10, and 11 of the 14 items, respectively, with a significantly higher PR for somatic symptoms in the poor teleworking environment group than in the good teleworking environment group. However, the only exception for a PR value significantly lower than 1 was for “working at a standing desk” for the cut-off of 12 (high severity) points, which had a PR of 0.90 (95% confidence intervals: 0.82-0.99), indicating a reverse association.

**Table 3:** Prevalence ratios of somatic symptoms according to telecommuting environment characteristics (all participants).

**Telecommuting environment characteristics**			**Somatic symptoms (the cut-off point)**												
			**Low (≥4)**			**Medium (≥8)**			**High (≥12)**			**Very high (≥16)**		
		***n***	**PR**	**95% CI**	***P***	**PR**	**95% CI**	***P***	**PR**	**95% CI**	***P***	**PR**	**95% CI**	***P***
**A place or room to concentrate**	Yes	3265	ref.			ref.			ref.			ref.		
	No	1304	**1.10**	**1.06-1.14**	**<.001**	**1.17**	**1.11-1.24**	**<.001**	**1.31**	**1.21-1.43**	**<.001**	**1.60**	**1.39-1.84**	**<.001**
**Sufficient desk light**	Yes	3805	ref.			ref.			ref.			ref.		
	No	764	**1.09**	**1.05-1.13**	**<.001**	**1.22**	**1.15-1.30**	**<.001**	**1.47**	**1.35-1.61**	**<.001**	**1.80**	**1.57-2.07**	**<.001**
**Sufficient workspace on the desk**	Yes	3332	ref.			ref.			ref.			ref.		
	No	1237	**1.10**	**1.06-1.14**	**<.001**	**1.18**	**1.12-1.25**	**<.001**	**1.39**	**1.27-1.51**	**<.001**	**1.56**	**1.36-1.78**	**<.001**
**Sufficient foot space**	Yes	3434	ref.			ref.			ref.			ref.		
	No	1135	**1.10**	**1.06-1.14**	**<.001**	**1.18**	**1.11-1.25**	**<.001**	**1.35**	**1.24-1.47**	**<.001**	**1.62**	**1.41-1.86**	**<.001**
**Comfortable temperature and humidity**	Yes	3689	ref.			ref.			ref.			ref.		
	No	880	**1.11**	**1.07-1.15**	**<.001**	**1.22**	**1.14-1.29**	**<.001**	**1.42**	**1.30-1.55**	**<.001**	**1.76**	**1.54-2.03**	**<.001**
**Quiet environment**	Yes	3272	ref.			ref.			ref.			ref.		
	No	1297	**1.13**	**1.09-1.17**	**<.001**	**1.20**	**1.13-1.27**	**<.001**	**1.36**	**1.25-1.49**	**<.001**	**1.63**	**1.42-1.87**	**<.001**
**Financial support for work environment improvement**	Yes	1473	ref.			ref.			ref.			ref.		
	No	3096	**1.11**	**1.06-1.15**	**<.001**	1.06	0.9997-1.13	.051	1.05	0.96-1.15	.255	1.03	0.90-1.19	.656
**Advice and guidance from the workplace**	Yes	1778	ref.			ref.			ref.			ref.		
	No	2791	**1.09**	**1.05-1.13**	**<.001**	**1.09**	**1.03-1.16**	**.003**	1.07	0.98-1.16	.215	**1.16**	**1.01-1.33**	**.039**
**Use of an office desk and chair**	Yes	2826	ref.			ref.			ref.			ref.		
	No	1743	**1.07**	**1.03-1.11**	**<.001**	1.05	0.995-1.12	.074	**1.23**	**1.13-1.34**	**<.001**	**1.36**	**1.19-1.56**	**<.001**
**Use of a low table or kotatsu**	No	2980	ref.			ref.			ref.			ref.		
	Yes	1589	0.98	0.95-1.02	.327	0.99	0.93-1.05	.700	1.04	0.95-1.13	.366	1.00	0.87-1.15	.992
**Use of a standing desk**	Yes	736	ref.			ref.			ref.			ref.		
	No	3833	1.04	0.994-1.09	.088	0.94	0.88-1.01	.073	**0.90**	**0.82-0.99**	**.025**	0.87	0.75-1.01	.071
**Stable communication environment**	Yes	3617	ref.			ref.			ref.			ref.		
	No	952	1.07	0.997-1.08	.068	**1.21**	**1.14-1.28**	**<.001**	**1.41**	**1.30-1.54**	**<.001**	**1.53**	**1.34-1.75**	**<.001**
**Adequate performance of communication devices**	Yes	3580	ref.			ref.			ref.			ref.		
	No	989	**1.04**	**1.001-1.08**	**.043**	**1.15**	**1.09-1.22**	**<.001**	**1.33**	**1.22-1.45**	**<.001**	**1.56**	**1.36-1.80**	**<.001**
**A place or environment to refresh oneself**	Yes	3273	ref.			ref.			ref.			ref.		
	No	1296	**1.08**	**1.05-1.12**	**<.001**	**1.13**	**1.07-1.20**	**<.001**	**1.24**	**1.13-1.35**	**<.001**	**1.39**	**1.22-1.60**	**<.001**

The PR was calculated for each cut-off for somatic symptoms (“Low” PR was classified as 0-3 points and 4-32 points, and PR of 4 or more points was calculated). Adjusted for sex, age, lifestyle behaviors (smoking, drinking alcohol, and physical activity), mental health, annual household income, education, type of industry, working hours, company size, and frequency of telecommuting. Each telecommuting environment was put into the model independently. Bolded figures indicate significant differences. PR, prevalence ratio.

Appendix 2 presents the results of the same analysis for the group with a telecommuting frequency of 3 or fewer times per week. Using cut-offs of 4, 8, 12, and 16 points for increasing severity of somatic symptoms, the PRs for having somatic symptoms were significantly higher for 12, 11, 10, and 11 of the 14 items, respectively, in the group with a poor telecommuting environment than in the group with a good telecommuting environment. However, the only exception of a significantly lower PR was for “working at a standing desk,” for the cut-off values of 8, 12, and 16 points, indicating a reverse association for this working condition.

Appendix 3 presents the results of the same analysis for the group that telecommuted at least 4 times a week. Using cut-offs of 4, 8, 12, and 16 points for increasing severity of somatic symptoms, the PRs for experiencing somatic symptoms were significantly higher for 7, 9, 10, and 10 of the 14 items, respectively, in the group with a poor telecommuting environment than in the group with a good telecommuting environment. However, a lower PR of 0.85 (95% CI: 0.75-0.95) for “working at a table or a kotatsu” was observed in the case of 8 points, indicating a reverse association.

## 4. Discussion

This study examined the relationship between teleworking environments and somatic symptoms among teleworkers. The results showed consistent findings that the PR of somatic symptoms increased significantly as the poor teleworking environment increased at both cut-offs of the Japanese version of the SSS-8 and teleworking frequencies. Similarly, for each teleworking environment, the somatic symptom PR was significantly higher for the following 6 items: a place or room to concentrate; sufficient foot space; comfortable temperature and humidity; quiet environment; stable communication environment; and a place or environment to refresh oneself.

The finding that a poor telecommuting environment was associated with a higher prevalence of somatic symptoms supports the findings of previous studies, which reported that shoulder pain^4^ and low back pain^5^ were associated with a poor telecommuting environment. In the present study, similar associations were found for several somatic symptoms, including shoulder pain and low back pain. In addition to ergonomic aspects, the occurrence of somatic symptoms could be due to stressors generated by the teleworking environment. Somatic symptoms can arise from anxiety, depression, common mental disorders, and stress-related disorders.^21^ A poor teleworking environment may be a stressor, causing various somatic symptoms as a stress reaction.

Furthermore, analyses stratified by the teleworking frequency showed similar associations for both those who teleworked 3 or fewer times per week and those who teleworked 4 or more times per week. In a previous study that found an association between teleworking frequency and low back pain, the odds ratio of having low back pain was significantly higher in the group that teleworked 2-3 days per week than in the group that rarely teleworked in poor teleworking conditions.^8^ Therefore, even with infrequent teleworking, a poor telecommuting environment may induce somatic symptoms.

Regarding individual telecommuting environments, 6 factors were associated with somatic symptoms in all analyses. One was the presence of a place or room to concentrate. A systematic review of home office set-ups and somatic health indicated that having a room dedicated to professional activities is associated with a lower likelihood of developing health problems.^6^ Additionally, a previous cross-sectional study has shown that having one’s attention diverted while teleworking is associated with more physical health problems.^13^ The present study results are consistent with previous study results. The blurring of the boundaries between work and home may explain why this association was observed. A review of teleworking and worker health and well-being suggests that teleworking blurs the boundary between work and home and that workers who can successfully manage this boundary are at a lower risk of adverse occupational health effects.^10^ Having a place or room to concentrate on work may make it easier to control this ambiguity.

The second environment associated with somatic symptoms in both analyses was the lack of sufficient foot space. This is consistent with previous research, which has shown that an inadequate environment is associated with shoulder pain^11^ and low back pain.^12^ The lack of legroom tends to result in an unnatural posture. A cohort study of Dutch computer office workers suggests that irregular head and body posture is a risk factor for neck and shoulder pain.^22^ Similarly, a cross-sectional study of Iranian workers showed that uncomfortable posture is associated with shoulder, hand, and wrist pain.^23^

Uncomfortable indoor temperature and humidity were associated with somatic symptoms in both analyses. A previous cross-sectional study has shown that dissatisfaction with the temperature and humidity of the telecommuting environment is associated with a higher number of physical health problems,^13^ and the present study result was similar. Inappropriate temperature and humidity were also associated with shoulder pain^11^ and low back pain,^12^ and the results were consistent. Possible explanations for these associations include cold and dryness, as those who frequently work in cold environments have reported more neck, shoulder, and leg pain.^24^ Dryness has been noted to cause eye and respiratory irritation, headaches, and fatigue.^25^

The requirement for a teleworking environment that is quiet, has a stable communication setting, and contains places and environments to refresh oneself to prevent somatic symptoms is the new finding of this study. These environmental factors are recommended for telework,^9^ lack of which may trigger a stress response in teleworkers, resulting in a variety of somatic symptoms, including musculoskeletal disorders. Teleworkers who work in poor environments are at a higher risk of musculoskeletal disorders.^26^

Conversely, no association was found in any of the analyses for the use of a low table, kotatsu, or standing desk with poorer PR for somatic symptoms. These findings were similar to those of a previous cross-sectional study that found no significant difference in the odds ratios for low back pain among teleworkers using a low table compared with those using a work desk/PC desk.^27^ The standing desk had the lowest sufficiency rate of 16.1% for the 14 environmental factors, suggesting that those with somatic symptoms, such as shoulder pain and low back pain, may have been aware of the prevention of aggravation and made the necessary environmental arrangements.

This study had a few limitations. First, as this was an internet survey, the selection of respondents may have been biased toward those with easy access to the internet, although most Japanese people have access to the internet. Second, because this was a web-based self-administered questionnaire survey, there is a possibility of some errors in the evaluation of telecommuting environments compared with objective evaluations. Third, because the design of this study was cross-sectional, there is a possibility of causal reversal. Future longitudinal studies should examine this association.

### 4.1. Conclusions

The results suggest that, among home-based teleworkers, the more inadequate the teleworking environment (especially places for concentration, foot space, temperature and humidity, quietness, communication environment, and change in mood), the higher the likelihood of somatic symptoms. Organizing these telecommuting environments with the aforementioned factors in mind may be useful in preventing various somatic symptoms. Future longitudinal evaluations are expected to clarify causal relationships using a higher level of evidence.

## Author contributions

S.K., T.T, and Y.K. conceived the idea; T.T. collected the data; S.K. analyzed the data and led the writing; T.T. and Y.K. reviewed the article and provided advice; all the authors have read and approved the final manuscript.

## Funding

This study (JACSIS2021) was supported by the Japan Society for the Promotion of Science (JSPS) KAKENHI grants (grant numbers 16KK0059, 18H03107, 19K10446, 21H04856); Health Labour Sciences Research Grants (grant numbers 19FA1012, 22JA0501); Innovative Research Program on Suicide Countermeasures (grant number R3-2-2); and Grants from Chiba Foundation for Health Promotion & Disease Prevention.

## Conflicts of interest

The authors declare no conflicts of interest for this article.

## Data availability

The data employed in this investigation are not accessible in a communal database as they encompass information that may identify individuals or potentially sensitive patient data. In accordance with the ethical principles governing research in Japan, the Research Ethics Committee of the Osaka International Cancer Institute has imposed limitations on the distribution of the data amassed during this study. All inquiries concerning the data should be directed to the individual accountable for data administration (T.T.) at tabuchitak@gmail.com.

## Supplementary Material

Web_Material_uiad014
